# Orthodontic Retainers—A Critical Review

**DOI:** 10.3390/children10020230

**Published:** 2023-01-28

**Authors:** Ioannis Lyros, Ioannis A. Tsolakis, Michael P. Maroulakos, Eleni Fora, Theodoros Lykogeorgos, Maria Dalampira, Apostolos I. Tsolakis

**Affiliations:** 1 Department of Orthodontics, School of Dentistry, National and Kapodistrian University of Athens, 11527 Athens, Greece; 2 Department of Orthodontics, School of Dentistry, Aristotle University of Thessaloniki, 54623 Thessaloniki, Greece; 3 Department of Oral Medicine & Pathology and Hospital Dentistry, School of Dentistry, National and Kapodistrian University of Athens, 11527 Athens, Greece; 4 “Hatzikosta” General Hospital of Messolonghi, 30200 Messolonghi, Greece; 5 Private Practice, 54124 Thessaloniki, Greece; 6 Department of Orthodontics, Case Western Reserve University, Cleveland, OH 44106, USA

**Keywords:** orthodontic treatment, maintenance of treatment result, fixed retainer, clear retainer, removable appliances

## Abstract

The achievement of aesthetic, functional occlusion should not mark the end of the orthodontic intervention. To prevent relapse, retention needs advance planning, and may vary in duration. This review aims to present and comment on the available means of retention. The ever-popular, passive Hawley-like removable appliances are credible in maintaining the desired occlusion. Modifications are the removable appliance Wrap Around, having the labial archwire extending to the premolars; the translucent retainer, Astics, a unique aesthetic Hawley-type device; and the reinforced removable retainer, which features a metallic grid reinforcing the acrylic base. Vacuum-formed retainers are easy to fabricate and are readily prescribed. By contrast, fixed retainers are made of orthodontic wire and composite resin bonded on the lingual or palatal surfaces of the anterior teeth. Patient-related variables need evaluation to select the appropriate retainer, while patients ought to realize the importance of retention and comply with offered guidance. Overall, the orthodontist is responsible for keeping the patient informed on the properties and the duration of retention, even before starting active orthodontic treatment.

## 1. Introduction

Following the conclusion of active orthodontic treatment, it is equally important to prevent relapse of malocclusion [1,2]. Nevertheless, the preservation of the therapeutic effect remains controversial in clinical orthodontic practice [3,4,5,6].

Establishing a desired dental occlusion as planned, results in disorganized periodontal fibers and newly formed bone, not yet fully adapted to the modified structure [7,8,9,10]. Tooth arrangement tends to relapse, gradually returning to its initial status [11,12,13]. The reason for the above-mentioned unwanted event remains partially understood, allegedly connected to the periodontal membrane, the occlusion, the surrounding soft tissues, and the overall growth [11,14,15,16,17,18].

Views and practices regarding retention vary as a result of a lack of robust clinical evidence and individual clinical experience [19,20,21]. The orthodontist takes into account the occlusal and craniofacial changes likely to emerge, the treatment plan, [22] the patient’s oral hygiene effectiveness, and oral habits, before implementing appropriate retention [2,23]. Retention can materialize either by removable or fixed appliances [11,22].

Removable Hawley-type devices, vacuum-formed retainers, lingually bonded wire, and their modifications are most commonly provided [4,24,25]. However, there is pervasive uncertainty on the optimal duration for their application [4], although it has been shown that regeneration of the periodontal apparatus may require up to 12 months to adequately support the tooth in its novel position [18,26]. Omitting dental retention, relapse is likely to occur eventually [27]. Therefore, most clinicians tend to apply long-lasting retention, even permanently [11,22]. Moreover, orthodontists may opt for periodontal fiber sectioning [28], labial frenectomy [29], and interproximal enamel reduction [30,31,32].

## 2. Hawley

**Figure 1:**
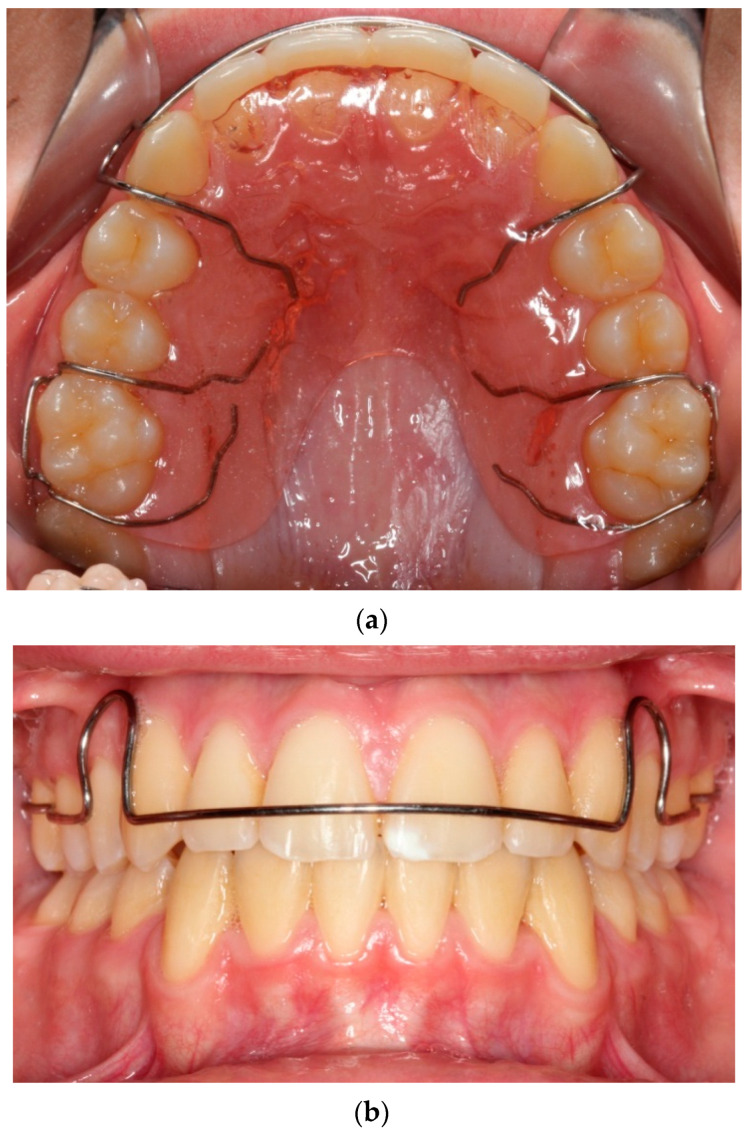

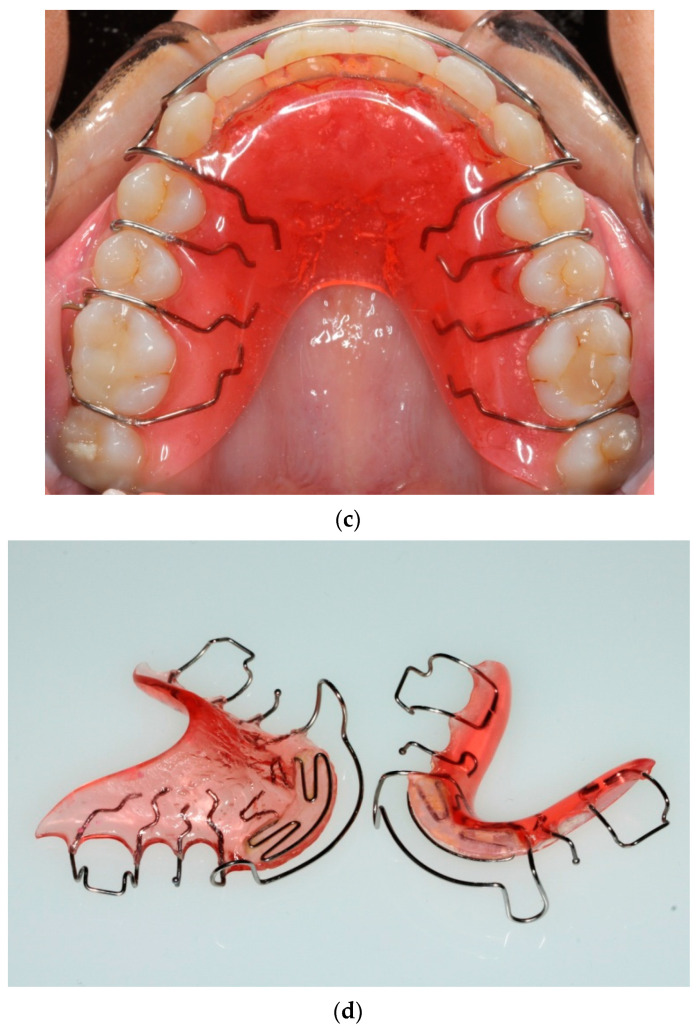

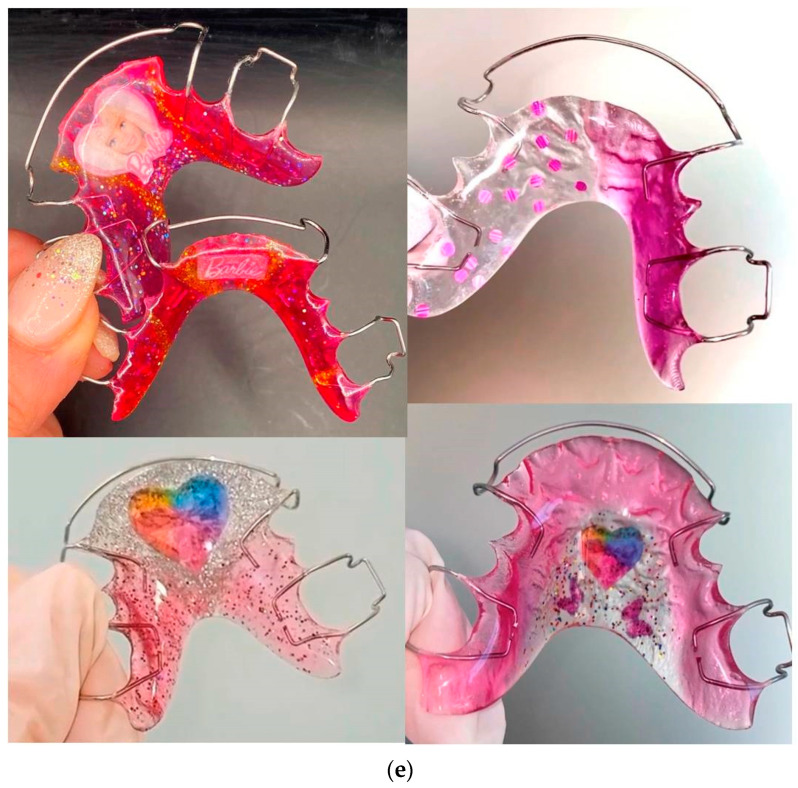
Hawley removable retainer. (**a**) Hawley occlusal view, (**b**) Hawley frontal view, (**c**) Hawley with ball slasps occlusal view, (**d**) Hawley with springs, (**e**) Hawley with colors and cartoon figures.

The Hawley appliance is the most popular removable retainer, fabricated of acrylic resin and wire. It consists of a labial archwire, clasps, and a palatal or lingual acrylic base (Figure 1a–c).

The stainless steel wire is cylindrical in cross-section, 0.28″–0.32″ in diameter. Carefully adapted to fit intimately on the labial surfaces of the anterior teeth (maxillary or mandibular), it has loops corresponding to the canines. The most commonly used clasp is of the Adams type, mainly applied around the first permanent molars, providing resistance to displacement. Alternative clasp types are the peripheral ones, easy to manufacture and gingivally friendly, but contraindicated in the cases of teeth with reduced clinical crown height and lacking anatomical shape due to the ensuing inadequate retention. Additionally, the ball clasps, most commonly fitted on posterior teeth (premolars), are in contact (Figure 1c). The acrylic base plate keeps wires and other components (e.g., screws, springs) in a proper position in relation to teeth. It needs to fit passively at the palatal or lingual gingival margins to provide tooth support, increasing retention [33]. Properly fabricated springs may be added to correct minor dental relapse (Figure 1d). Adding colors and cartoon figures makes the appliances friendlier for younger patients and may even improve their compliance (Figure 1e).

## 3. Astics Translucent Labial Bow (ATLB)

**Figure 2:**
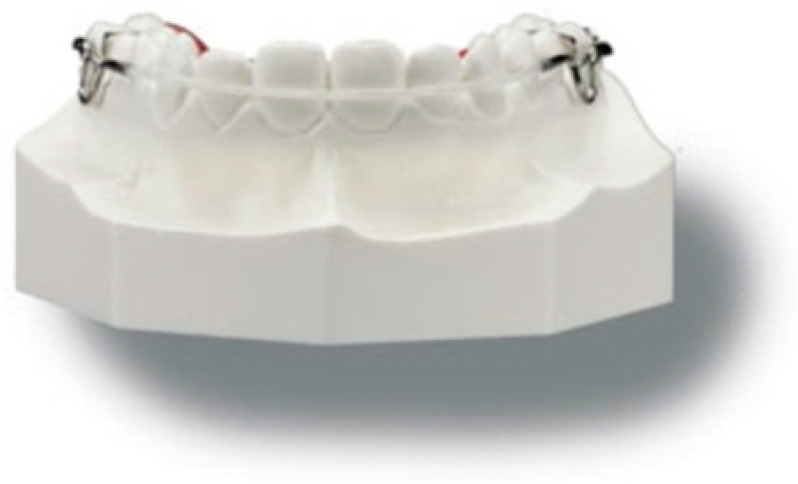
Astics translucent labial bow.

Despite its popularity among orthodontists and the attempt to improve aesthetics, the Hawley retainer is still imperfect in appearance because of the prominent labial metallic wire. A breakthrough to this deadlock might be the ATLB (Figure 2). The Astics bow is semi-solid, manufactured of fiber-reinforced composite resin, fitting into a tube welded to the Adams clasps. Adams clasps wrap around the first permanent molars to provide retention. There are metallic connectors and also an acrylic base plate. Contrary to the Hawley, the Adams clasps are welded to metallic connectors, and they also support the Astics bow. The wire assumes its final shape on the working model, light-cured in contact with labial dental surfaces.

The ATLB is the only aesthetic solution for the provision of a retentive appliance resembling Hawley because the transparent labial resin is barely noticeable. The bow is not inferior in durability because fibers offer considerable fracture resistance, while being stable in color as they do not absorb pigments, being totally embedded in the resinous mass [34].

## 4. Wrap Around (WA)

**Figure 3:**
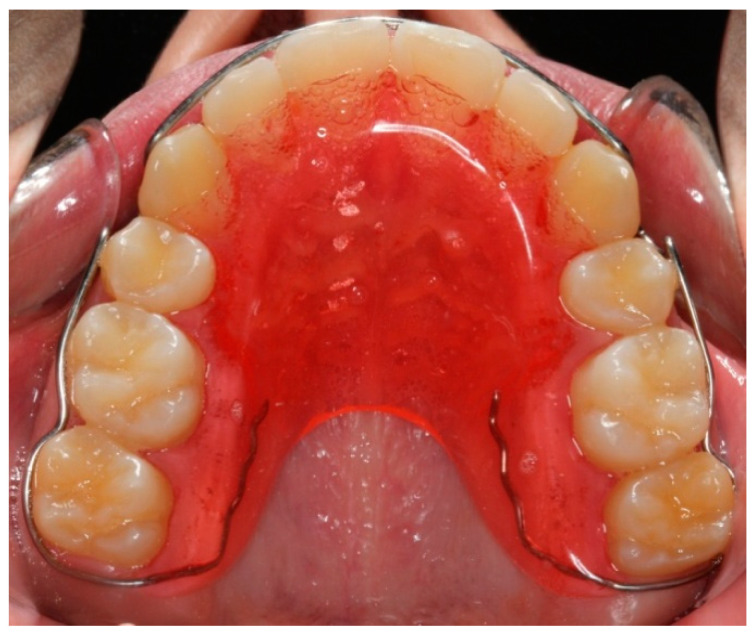
Wrap Around removable retainer.

In the WA removable appliance (Figure 3), the labial archwire extends to the posterior teeth, encompassing the premolars, with the absence of molar retentive clasps [35]. The device is suitable in cases where the treatment plan includes extractions. On the other hand, the elongated wire may end-up prone to distortion, and is further undermined by mishandling during fitting and removal. Patients are advised to remove the appliance from the palatal acrylic using their thumb or forefinger, while experienced patients may also use their tongue. It is advisable to place acrylic on the labial bow to increase stability and prevent potential distortion. Some clinicians use the appliance as an alternative when the classic Hawley is blamed for occlusal interference.

## 5. Reinforced Removable Retainer (RRR)

The RRR is a modification of the well-known Hawley appliance, being reinforced with metallic mesh, and also has ball clasps. The mesh adds resistance to breakage without adversely affecting soft tissue health, since it is fully integrated into the body of the acrylic. The mesh is kept extremely thin to facilitate handling during manufacturing, and to prevent the appliance thickening and becoming uncomfortable and unacceptable. Additionally, ball clasps increase retention and stability [36]. Due to its limited application, a further clinical investigation is needed to draw reliable conclusions.

## 6. Invisible Thermoplastic/Vacuum-Formed Retainers (VFR)

**Figure 4:**
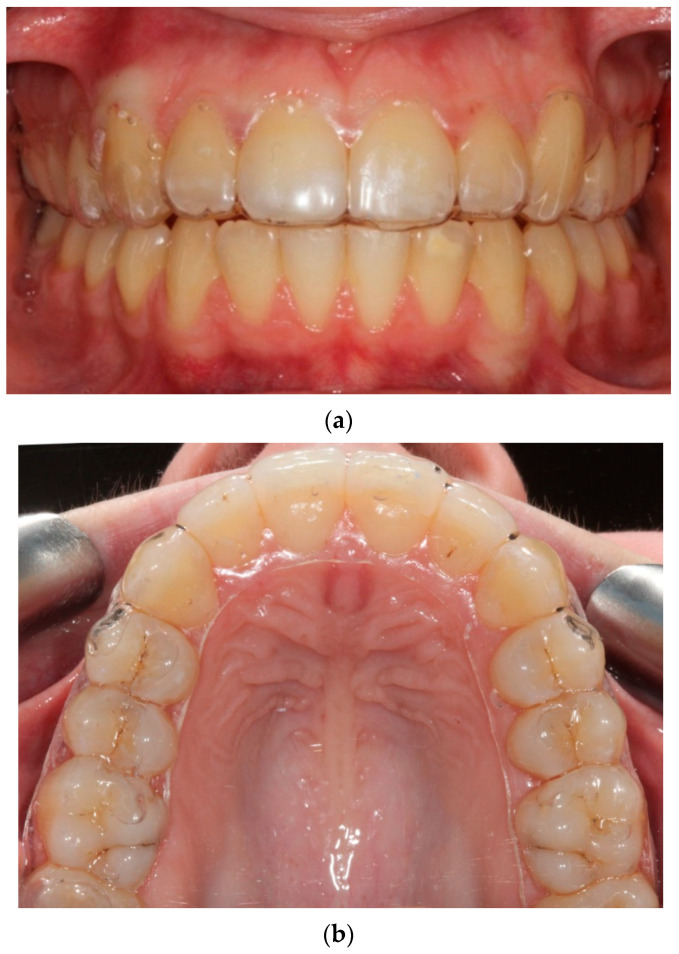
Vacuum-formed retainer. (**a**) Frontal view, (**b**) Occlusal view.

In 1971, Ponitz [37] introduced the Thermoplastic Stabilization Splint as an alternative to the existing ordinary removable device (Figure 4a,b). A thermoplastic sheet (polyethylene terephthalate glycol copolymer, 0.040″ in thickness) is heated and compressed inside a vacuum apparatus against the patient’s mold, according to the manufacturer’s instructions, to then be trimmed into a horseshoe shape [33]. The thickness of the thermoplastic should be properly selected for reasons of patient comfort and increased durability.

The VFR is aesthetically pleasing, easy to clean with soap and water, and costs only a fraction of the conventional Hawley [38]. It usually requires no adjustment when fitted [39] and many clinicians find that it is more acceptable by patients due to its superior appearance and easiness of application [40,41]. In addition, the retainers are quick and easy to fabricate in the dental lab or in-house, just a few materials are required [39,42]. Overall, only limited technical skill is necessary because wire bending does not apply. Nevertheless, compared to Hawley, it appears to wear out more easily, and may not always prove to be dimension-stable, which might jeopardize the long-term stability of the treatment outcome. Furthermore, the thermoplastic rests between occluding teeth surfaces and so it prevents vertical tooth movement and subsequent occlusal adjustment [38,43].

## 7. Positioner

**Figure 5:**
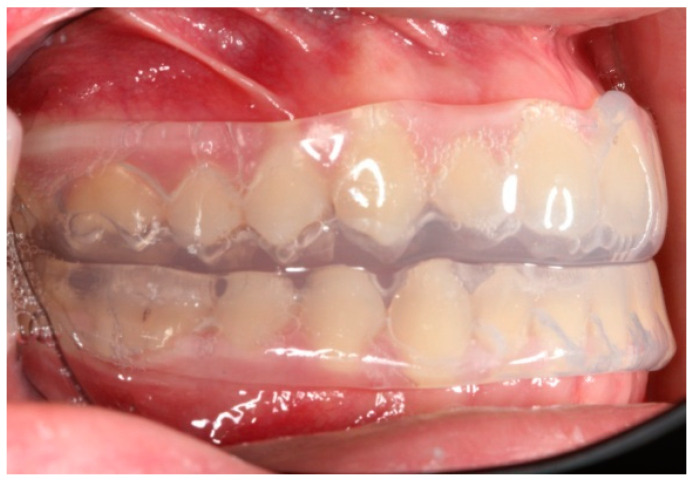
Positioner.

It is a transmaxillary, removable appliance, considered one of the most effective retention devices ever invented. It is custom-made, fabricated in the lab from resilient translucent silicone (Figure 5) with the aid of a cast with teeth that have been properly set-up. It has also been used to correct minor intra- and inter-arch irregularities in cases where active treatment needs to cease prematurely. It may cause minor, programmed tooth movement, while respecting the gingival tissues. However, long-term compliance is questionable because some patients consider it unaesthetic, bulky, and dysfunctional. However, it could allow for earlier completion of active treatment, promote small space closing, and minor rotation or buccolingual correction. It could even alleviate some occlusal discrepancies. It improves lip competence and facial muscular tone. It could be used to correct second molar crossbites, and to control overjet. However, it is not possible to achieve more than 1–2 mm of respective tooth tipping [44,45].

## 8. Fixed Retainer

Numerous researchers advocate for the necessity of applying permanent retention, with the aim of avoiding relapse in the distant future [46,47,48,49]. In such cases, fixed retainers prove to be the most efficient. Various techniques have been introduced, the most popular combining orthodontic wire with composite resin. It constitutes best practice after correcting anterior tooth crowding. It can be applied to both the upper and lower anterior segments, but it is electively avoided in the maxilla as it may interfere with occlusion and mastication.

**Figure 6:**
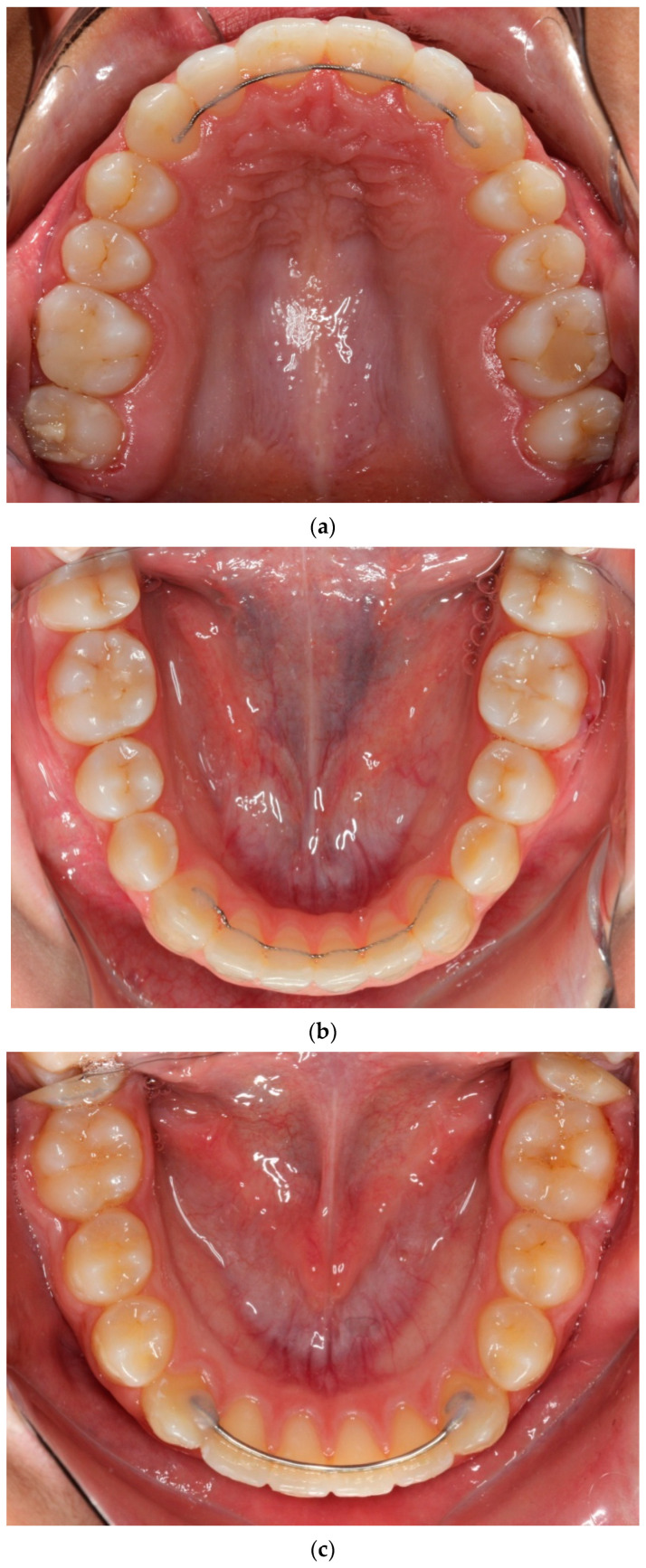

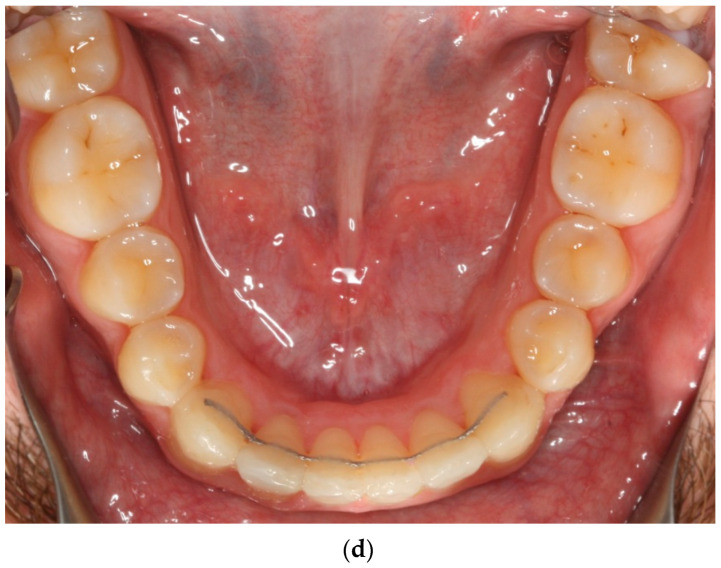
Fixed permanent retainer. (**a**) Upper canine to canine flexible cylindrical wire, (**b**) Lower canine to canine flexible cylindrical wire, (**c**) Lower canine and canine non flexible cylindrical wire, (**d**) Lower canine to canine flattened wire.

Two alternatives have been used, namely an intercanine lingual/palatal wire bonded on all six anterior teeth (canine to canine) (Figure 6a,b), or lingual/palatal intercanine wire bonded only to the canine lingual/palatal surfaces (canine and canine) (Figure 6c) [50,51,52]. The latter alternative is regarded as more effective in preventing changes in the intercanine dimension, but may not keep the dental units in their designated position, potentially culminating in future crowding [53]. The wire required is 0.30″ of stainless steel, and there is a need for sand-blasting the canines’ lingual surfaces.

When bonding only to the canines, dental enamel may need to be removed from the interproximal tooth regions to create contact surfaces instead of points, with the aim of enhancing stability. The above practice might prevent micro-rotation, but not totally prevent bodily tooth movement.

According to the research of Störmann and Ehmer (2002), splinting of all six anterior teeth causes minimal patient discomfort, but increased rates of resin debonding may emerge regarding any of the teeth [53]. This often remains unnoticed by the patient, in contrast to the canine and canine variant, a common occurrence calling for compulsory annual patient follow-up [54].

Flexible stranded wire, cylindrical (Figure 6a,b) or flattened (Figure 6d), is the most commonly used. It may be manufactured from steel or alloys containing chromium, nickel, gold, and titanium. A CAD/CAM procedure may prove a safe and reliable option. Indeed, less plaque and gingival inflammation have been associated with CAD/CAM retainers [55]. It is used mainly to prevent a relapse of dental spacing and rotation and to reduce potential secondary crowding. The twistflex stainless steel wire has the property of allowing for normal dental micro-movements within the alveolar socket, but patients may call with unwanted tooth movement, to such an extent that retreatment is warranted [56]. An increase in the wire diameter potentially alleviates the prognosis of occlusal stability. However, Gökçe and Kaya [57] failed to correlate the success rate of fixed retention to the thickness of the wire. This is in agreement with the studies of Baysal et al. (2012) [58] and Al-Nimri and Al-Nimri (2015) [59]. Therefore, considering the existing evidence, it is wise not to attribute any potential failure to the diameter of the wire being used.

A variant of the fixed retainer integrates fiberglass fibers into flowable composite resin bonded to the anterior lingual tooth surfaces [60]. Comparing the two types of fixed retainer, it appears that the retainer with the metal wire promotes less plaque accumulation, it may allow for easier oral hygiene performance, may be blamed for less severe gingivitis [61], and has lower failure rates [62]. There is no agreement on whether the properties of the wire contribute to significant differences in the clinical performance or the maintenance of healthy tissues [63,64,65,66]. Fixed retention requires patient cooperation in applying daily, effective oral hygiene with mechanical (tooth brushing, interdental cleaning with interdental brushes or superfloss) and chemical (mouthwashes) means. Cooperation should be anticipated in the case of removable retainers, which do not equally impact the periodontium.

## 9. Discussion

There is inadequate evidence to suggest that fixed retention systems are more effective in maintaining the results of orthodontic treatment. Fixed retainers are more effective at maintaining incisor alignment during the first semester, [67] but there is no statistically significant long-term difference between fixed and removable devices regarding irregularity indices, the intercanine or intermolar distances or the arch length, and the fate of post-extraction spacing [23,68]. In particular, Artun et al. (1997) [69] and Littlewood et al. (2004) [70] found no significant differences after comparing different fixed and removable retention protocols.

Only relative indications may be considered for any particular mean of retention. The selection should be individualized because the risk of recurrence and other factors differ between patients. In essence, success in retention relates to the degree of communication and cooperation with the patient [71]. Evaluating the patient profile and the feedback throughout the active phase of orthodontic treatment contributes to a more feasible application of an effective retentive appliance. Indeed, it is particularly important that the patient becomes informed in advance that treatment does not end with appliance removal, but proceeds with the equally important stage of preserving the therapeutic result [72]. Hence, the orthodontist should spend time explaining the importance of the above-mentioned procedure, providing motives in the right direction so that the outcome does not become imperiled [2].

Thickett and Power (2010) [73] and Jaderberg et al. (2012) [74] did not report differences in effectiveness regarding the duration of use of the VFRs (part-time versus full-time). This is in agreement with Gill et al. (2007) [75] and Lindauer and Shoff (1998) [76]. Still, there is no established retention protocol for VFRs [77]. Overall, evidence of high-quality indicates that part-time VFR application is probably equally effective compared with full-time use [70,78,79]. Thus, it would seem reasonable to accept that these retainers could be prescribed for night-only use. Part-time wearing of the VFR might also be related to the increased longevity of the material. On the other hand, full-time application could be associated with greater failure rates [80]. Similarly, Sawesh et al. (2010) [81] found no significant difference between part- and full-time Hawley retainer use and so they suggested that the orthodontist should prescribe nighttime-only use of the Hawley, lasting for one year, immediately after the conclusion of active treatment. Comparing different retention protocols, Edman Tynelius et al. (2015) [82] found that all the techniques of interest (fixed maxillary and mandibular retention with tooth stripping, Positioner) can prove effective in stabilizing the dentition [83].

Removable devices require increased cooperation and consistency on the patient’s side regarding maintenance and application [84,85], otherwise a fixed alternative might prove a better option. Hawley-type appliances, combining an acrylic base plate and wire arch, are considered an optimal functional solution to retain the entire dental arch [86,87]. The risk of caries may increase only in cases of inadequate oral hygiene practice [69,88,89], as patients with orthodontic retainers have been found probably more vigilant with tooth cleaning [90]. Indeed, the favorable effect of fluoride toothpaste, mouthwash, and other products may prove more pronounced in people with fixed retainers because of the ensuing increase in oral fluoride retention [91]. Al-Kuwari et al. (2015) [90] and Gupta et al. (2017) [88] noticed that the accumulation of bacterial plaque around the bonded lingual wire did not cause a statistically significant increase in tooth decay. Additionally, they found that fixed retention was not a serious obstacle to effective dental plaque removal. On the other hand, cases of caries in patients wearing VFRs have been related to only partial compliance with instructions, and to cariogenic eating practices [92,93]. Hence, orthodontists and general practitioners should assume the duty of empowering patients on oral hygiene practice, educating on the risk of demineralization, and reminding about frequent, preventive visits [90].

Artun et al. (1997) [69] alleged that plaque accumulation around bonded retainers may not be such an important issue to seriously affect periodontal health. Nevertheless, Rody et al. (2011) [94] and Rody et al. (2016) [95] registered alterations in gingival crevicular fluid composition and they considered them as indicating insidious inflammation related to the restraining effect exerted by the appliance on the anterior and posterior mouth areas.

Salvesen et al. (2021) [96] concluded that prolonged fixed retention per se does not have harmful periodontal outcomes, but coexisting factors such as smoking and hand dexterity may increase the risk of plaque accumulation causing inflammation. In fact, their sample self-reported that they felt confident for effective oral hygiene. Fixed retainers have been associated with a greater accumulation of dental plaque and calculus, and with minimally worse, albeit clinically unimportant, gingivitis in comparison with VFRs [97]. Moreover, patients using Hawley appliances may end up in an even better periodontal condition compared with those using VFRs [98]. Eroglu et al. (2019) [99] found fixed and removable orthodontic retainers not statistically significantly different regarding the plaque index, the gingival index, bleeding on probing, and probing depth values. In addition, they observed that oral hygiene improves only after the debonding of the fixed appliances. Arn et al. (2020) [100] suggested that fixed retainers seem suitable even for patients with compromised periodontal health because detrimental consequences are not very likely to arise.

Particular wire properties do not appear to be contributory to any noteworthy deterioration of the periodontium [101]. According to Bucur et al. (2022) [102], plaque accumulation is significantly lower in removable compared to fixed retainer bearers, but in Hawley appliance wearers, interdental plaque may also be prominent. Not surprisingly, gingival recession was found prevalent around fixed retainers. Fiberglass appears inferior compared to braided wire due to its increased thickness and the frequent delamination, which can end up being embarrassing [60]. The significance of patient support regarding effective oral hygiene maintenance should be well-established [103,104].

Potential future relapse after the end of active treatment or the plan for future intervention (e.g., extractions, indicated orthognathic procedures) are equally important factors to consider when choosing a retainer. Residual growth, skeletal or dental, directly related to the patient’s chronological age [14,15,16,17], and muscular activity need to be carefully appraised [105]. For example, Hawley retainers with anterior or posterior bite plates are indicated in patients having a deep bite or open bite occlusion, respectively, at initial presentation. As a matter of concern, aesthetic requirements are highly subjective, depending on the patient’s social and professional activity.

Post-orthodontic treatment patients having fixed retainers bonded on their anterior teeth might need a magnetic resonance imaging (MRI) evaluation. It has been claimed that the diagnostic quality of the images might deteriorate due to the distortion caused by the potent magnetic field, especially if the metallic orthodontic appliance adjoins the area of interest [106,107]. Stainless steel, cobalt, and chromium cause significant artifacts, rendering several cranial regions difficult to diagnose [107,108,109,110,111]. For example, twistflex retainers, made of stainless steel wire, have been evaluated in vitro, causing unacceptable artifacts in the diagnostic images that extend even extraorally. However, Beau et al. (2015) [112] claimed that steel fixed retainers cause poor image quality only intraorally and that they need to be removed ahead of an MRI only if the area under investigation is inside the oral cavity. CAD/CAM retainers, too, may cause substantially smaller distortion and, thus, they are likely to have less pronounced effects on image quality [111].

According to Shalish et al. (2015) [113], it is not necessary to remove twistflex and gold-nickel fixed retainers before MRI scans. Removal might be considered only if the examination aims at the jaws or the tongue and retainers are bonded at both the maxilla and the mandible. If the region of interest is further away from the fixed retainer, there is virtually no need to remove it. However, fixed retainers having unacceptable ferromagnetic properties might need to be removed prior to MRI scanning [107,109]. Additionally, patients having a retainer made of polymer or ceramic materials, gold, or titanium must be carefully evaluated [114], although the above materials do not seem to pose a significant risk of adverse body effects [106] or image distortion [107,115]. Ideally, orthodontists could consider avoiding retention with stainless steel wire or metallic brackets in patients who are likely to require frequent MRI investigations [107].

In patients with maxillofacial dysfunction complicated by occlusal irregularity, the most balanced occlusal alignment must be sought and, therefore, the goal should be to provide credible retention. In case of allergy to acrylic, the Hawley might be substituted either by a fixed retainer or by the use of a Hawley-type appliance with palatal thermoplastic or an alloy, despite the concomitant discomfort due to thickening. Although it is feasible to unveil the eventuality of allergic reaction to acrylic by skin patch testing [116], it is not an ordinary procedure readily available for most patients, except in cases of proven history or a strong suspicion of hypersensitivity. More rarely, mucosal lesions may be more challenging to diagnose because they imitate common oral inflammatory lesions. Nevertheless, the disturbing immune response readily resolves after the removal of the cause [117].

Fixed retention is effective and reliable, the gold standard in post-orthodontic mandibular anterior stability. However, composite bond failure is the most common problem and it has been associated with the individual shape of the mandibular incisors’ lingual surfaces [118,119,120]. Aiming to address this occurrence, different ways for stabilizing the composite and wire have been introduced, namely direct and indirect [121]. Indirect bonding is considered challenging because it involves trays to apply consistent, steady pressure, and thus it might culminate in an uneven retainer structure and, thus, inadequate bond strength [122]. Nevertheless, it may prove advantageous in terms of moisture control while manipulating the device [123,124]. On the other hand, the working field in direct bonding is clearer, contributing to the reliable control of the composite resin polymerization and adhesion [125]. Interestingly, though, the periodontal effects and the success rates of the above methods are rather comparable.

Reportedly, the incidence of failing fixed retainers appears greater during the first six months following placement [126,127]. In fact, Egli et al. (2017) [119] and Taner and Aksu (2012) [120] claimed that the majority of failures may be expected within the first month of bonding. Additionally, Gökçe and Kaya (2019) [57] noted several detachments occurring during a three-month follow-up, progressively decreasing thereafter. Despite the increased rate of failure immediately post-bonding, an increase in bond failures might also be anticipated throughout the entire observation period, as has been demonstrated by Lie Sam Foek et al. (2008) [126].

Fixed retention appears more reliable compared with removable retention for keeping incisors aligned during the initial semester of retention. However, there is virtually no known difference in the outcome between the various known fixed retention systems. For example, within the limitations of the present review, we wish to suggest that there does not seem to exist any remarkable difference in the failure rate between the direct and indirect bonding techniques of mandibular 3 × 3 fixed retainers. Similarly, there is no difference in efficacy between the different removable retention systems. The part-time use of removable retainers (approximately 10 h/day) suffices to maintain the outcomes of orthodontic treatment. Thus, there is insufficient evidence to consider adopting a particularly effective retention protocol. For this, prospective randomized clinical trials would be warranted. Similarly, prospective studies are required to evaluate the long-term risks and behavior of the various retention systems, and their impact on periodontal integrity in particular [83]. From a clinical perspective, the decision-making procedure regarding retainer selection should involve variables such as the cost–benefit, the upper–lower arch discrepancy, the orthodontist’s preferences, the subjective impact on the quality of life, and, more importantly, the level of patient compliance and motivation. It is necessary to remember that post-orthodontic appointments for treatment stability assessment are also an essential part of orthodontic treatment. The patient should be followed up regularly after the removal of fixed appliances, independently of the retainer of choice.

Failure of fixed retention is likely to culminate in the relapse of crowding. Kučera and Marek (2016) [128] estimated the rate of complications regarding mandibular bonded multistranded retainers as being 1.1%. Most of the complications occurred within the first 5–6 years after bonding the retainer. Allegedly, the event cannot be fully attributed to insufficient passivity during placement or to the type of wire used or to the degree of incisor maladjustment [128]. More serious accidents with retainers are quite uncommon, but the orthodontist and the general dental practitioner should be vigilant for the likelihood of occurrence [129]. Ingestion [130,131] and aspiration [132,133] have been reported to require conservative [130,131,132] or more invasive surgical intervention [133,134] to correct, and upsetting complications also arose [131,132,134]. Overall, these incidents should be considered emergencies that must be quickly addressed and admittance to the hospital should be an option. Retrieval of the impacted objects of dental origin may be performed by endoscopy [131,132].

Long after the end of active treatment, fixed retention has been blamed even for severe tooth dislocation with gingival recession and buccal bone dehiscence that could have compromised tooth vitality and survival [135]. Similarly, the severe periodontal loss of attachment that ultimately required periodontal surgical correction has been attributed to the initial incorrect adaptation of the fixed mandibular retainer [136]. Therefore, patients having post-orthodontic treatment retention should regularly visit their general dental practitioner (or even the orthodontist) and the orthodontist should be available to deal with complications in case they emerge.

It has been suggested that the flexible twisted retainers have the potential to produce inadvertent tooth displacement [136]. Engeler et al. (2021) [137] demonstrated that plain and braided retainers transfer torsional loading more evenly in comparison to the multistranded ones, which are suspected of storing more energy to induce a subsequent unexpected tooth dislocation. Additionally, the impact of chewing forces activating a previously passive retainer should be a matter of concern [58]. However, not all occlusal post-treatment deviations should be attributed to issues connected with retention. The emergence of late tooth crowding might stem from the process of active orthodontic treatment itself. Intercanine expansion and mandibular incisor protrusion, for example, may increase the risk of secondary crowding [138]. Therefore, this is the reason for prescribing both removable and fixed retention in cases where dental arch expansion has been performed [139].

Evidence in the recent systematic review by Bellini-Pereira et al. (2022) [77] suggests that mandible, fixed retainers maintain the treatment result more predictably during the initial 6 months of retention compared with VFRs, but after the first year post-treatment, there is a tendency for all major retention protocols to be equally effective. Nonetheless, in the long term, bonded retainers seem to prevail over the VFRs regarding their obvious retention capacity. Maxillary retainers are generally effective, preserving the results of the orthodontic intervention. The clinician must stay informed that bonded wires are often blamed for greater plaque and calculus accumulation than VFRs, at least initially. Longitudinally, both retainers are associated with a negative periodontal condition, highlighting the importance of optimal post-orthodontic oral hygiene practices. Retainers seemingly present similar failure rates in the upper arch during the first year of retention. Later, VFRs fail more often in the upper arch than bonded retainers. In contrast, fixed retainers present greater failures in the mandible. It is noteworthy that according to studies performed in various countries worldwide, the most popular retention protocol requires a VFR or Hawley retainer in the upper arch and fixed retention in the lower [83].

The results of this review should be interpreted with caution. Even though the studies included were well-selected according to their methodology, it should be highlighted that their findings may be affected by differing initial malocclusion, the degree of tooth movement, the age of the respective samples, the length of time wearing the VFR, the different materials for the fabrication of fixed retainers, among other factors that are related to the unexpected emergence of relapse, and that are, in fact, beyond the scope of the present review [70].

Concluding, the financial aspect is an emerging issue when selecting the means of retention. The expense is affected by the materials and the technique to be used, and may increase due to potential lab fees. Most patients seek orthodontic treatment mainly for aesthetic reasons [140,141]. Allegedly, patients with functional irregularity considered both the cost of treatment and the expected duration of therapy as likely barriers for orthodontic intervention [140,141]. Orthodontists and patients might even be interested in paying additional fees for non-invasive procedures to optimize the long-term stability of the treatment’s final outcome [142,143]. Hence, it could be hypothesized that a reasonable financial burden should not be a decisive determinant when deciding on the appropriate orthodontic treatment course.

## 10. Conclusions

Considering the evidence presented in the present review, the following conclusions may be reached:

- There is inadequate evidence to suggest which way of retention is more effective in maintaining the result of orthodontic treatment. Only relative indications may be considered for any particular mean of retention.
- The selection should be individualized because the risk of recurrence and other factors differ between patients. Fixed and removable retention alike can prove effective in stabilizing the dentition.
- The risk of caries and periodontal deterioration increase in the case of inconsistent oral hygiene practice and unhealthy diet.
- Success in retention is related to the degree of communication and cooperation with the patient.
- Orthodontists should assume the duty of empowering patients on oral hygiene practice and conducting frequent, preventive appointments.
- The results of this review should be interpreted with caution because malocclusion is affected by differing variables.

## Data Availability

Not applicable.
